# Barriers to antenatal syphilis screening in Burkina Faso

**DOI:** 10.11694/pamj.supp.2014.17.1.3423

**Published:** 2014-01-18

**Authors:** Fadima Yaya Bocoum, Seni Kouanda, Christina Zarowsky

**Affiliations:** 1 Département Biomédical et Santé Publique, Institut de Recherche en Sciences de la Santé, Ouagadougou, Burkina Faso; 2 School of Public Health, University of Western Cape, Cape Town, South Africa; 3 Institut Africain de santé publique, Ouagadougou, Burkina Faso

**Keywords:** Screening, syphilis, barriers, antenatal, Burkina Faso

## Abstract

**Introduction:**

Despite advances in treatment and management, syphilis remains a major public health problem in Burkina Faso. Syphilis in pregnancy poses major health risks for the mother and the fetus and also increases the risk for HIV transmission. Despite its potential benefits, antenatal syphilis screening is often poorly implemented in many sub-Saharan African countries. The purpose of the study is to identify and understand barriers affecting health system performance for syphilis screening among pregnant women in Burkina Faso.

**Methods:**

We conducted in-depth interviews and observations in the Kaya health district, Burkina Faso. Participants were purposively selected to capture a range of perspectives across different actors with different roles and responsibilities. Seventy-five interviews were conducted with health providers, district managers, facility managers, traditional healers, pregnant women, community health workers, and Non-Governmental Organizations (NGO) managers. Interviews were transcribed and organized into codes and categories using NVivo software.

**Results:**

Participants identified multiple barriers at health providers and community levels. Key barriers at provider level included fragmentation of services, poor communication, low motivation for prescription, and low awareness of syphilis burden. Cost of testing, distance to laboratory and lack of knowledge about syphilis were identified as barriers at community level.

**Conclusion:**

The study highlights barriers such as distance, cost of testing, and knowledge about syphilis. The introduction of point of care testing for syphilis could be an entry point for improving coverage of antenatal syphilis screening.

## Introduction

Despite several advances in treatment and management, syphilis remains a major public health problem. The World Health Organization (WHO) estimates that there are twelve million new cases of syphilis worldwide each year [1]. Ninety percent of syphilis cases occur in low income countries [1] and the prevalence ranges from less than 1% to 10%. African studies show prevalence during pregnancy of 2% in Mali [2], 3% in Nigeria [3], 5% in Botswana [4], and 7.3% in Tanzania [5]. In Burkina Faso, Kirakoya-Samadoulougou et al found a low prevalence of syphilis during pregnancy at national level but with important regional variations [6]. For instance, in Kaya District the prevalence was 7.5 in 2009 whereas in Ouagadougou it was 1% [7].

Syphilis in pregnancy poses major health risks for the mother and the fetus and also increases the risk for HIV transmission [8]. The World Health Organization (WHO) estimates that two million pregnant women each year are infected with syphilis globally [2]. The risk of vertical transmission could be up to 80% in early latent syphilis [2]. Approximately 1.2 million pregnant women with syphilis transmit the infection to their newborn every year [9]. It is estimated that 492 000 infants in sub-Saharan Africa die annually from congenital syphilis [10]. In Tanzania, a clinic-based study found that a quarter of women with high-titer active syphilis infection had stillbirths compared with 1% among seronegative women [11].

Maternal syphilis is detectable by serological screening and entirely treatable with penicillin. Therefore, screening and treatment for syphilis has been recommended as a routine part of antenatal care [12, 13]. In Burkina Faso, syphilis screening is recommended for premarital tests and during pregnancy [14]. Unfortunately, antenatal syphilis screening is often poorly implemented in many sub-Saharan African countries [15]. Currently, only 30% of women with syphilis are screened and treated in developing countries [16]. The influence of health systems issues on timely prenatal syphilis screening has been observed in several countries, including Bolivia, Kenya and South Africa [17]. In West African countries such as Burkina Faso, barriers to syphilis screening are understudied.

In this study, we sought to identify and understand barriers affecting health system performance for syphilis screening among pregnant women in Burkina Faso. Existing literature on syphilis screening among pregnant women suggests that antenatal care (ANC) is the cornerstone for the control of maternal syphilis. Thus, factors affecting attendance to ANC are likely to affect syphilis screening for pregnant women. We therefore explored various factors at policy, health provider, patient, and community levels that are likely to drive syphilis screening levels.

## Methods

### Study design

We conducted a Multilevel Assessment (MLA) [18] comprising of qualitative interviews and observations, as well as a review of existing data. For the latter, we assessed health information systems records, policy documents, service provider guidelines, training manuals, monitoring and evaluation reports and other relevant research reports and published literature. These data enabled us to investigate how the syphilis screening policy was implemented at facility level, the available indicators of its health outcomes, and any documented barriers to its implementation to date. The in-depth interviews were held with health providers, district managers, facility managers, traditional healers, pregnant women, community health workers, and representatives of national and international Non Governmental Organizations (NGOs) which work on maternal and child health issues to explore barriers and constraints which affect the effective delivery of maternal syphilis screening. During data collection, the first author also observed interactions between health workers and clients in selected health facilities.

### Study setting

**Figure 1:**
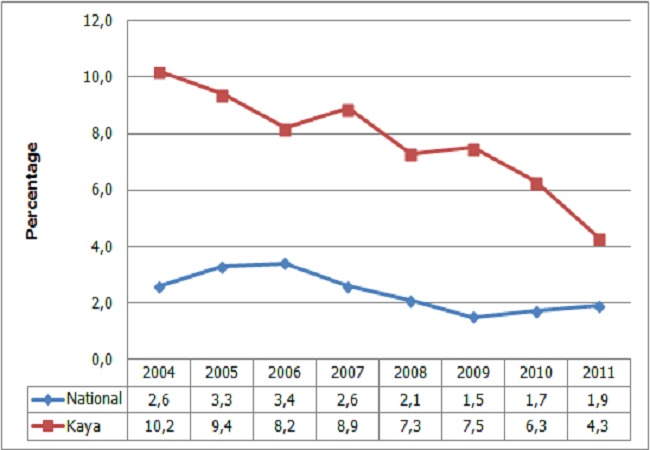
Trends in syphilis prevalence from 2004 to 2011 in Burkina Faso. Source: report CMLS/Santé, 2010, 2011

The study was conducted in the Kaya health district, based in the central north region of Burkina Faso. Kaya district has 484 932 inhabitants, 40 primary health facilities and is a sentinel site for the national AIDS and STI control program. We conducted this research in Kaya District because of the high syphilis prevalence relative to the national average. Figure 1 presents the trend of syphilis prevalence among pregnant women from 2004 to 2009 in Kaya district and nationally. The study was nested in the Kaya Health and Demographic Surveillance System (Kaya HDSS), which was launched in 2007 by the Health Sciences Research Institute (IRSS). Kaya HDSS covers seven semi-urban areas and 18 villages of the district with a population of 48,131 inhabitants. In 2011, there were seven public primary health facilities that offered ANC, one faith-based health center and one regional hospital. The faith-based facility and the hospital did not offer ANC but their laboratories offer the venereal diseases research laboratory (VDRL) test and Treponame pallidum hoemagglutination assay (TPHA). One pharmacy offered a rapid test for syphilis. The health facilities selected for the study were all located within Kaya HDSS area.

### Study population and sampling

The study population consisted of health providers, district managers, facility managers, traditional healers, pregnant women, community health workers, and NGO managers (Table 1).

**Table 1:** Study Sample

Group	Number and position
**District managers**	1 pharmacist
	1 information systems specialist
	1 reproductive health specialist
	Head of District
	Head of regional laboratory
**Health workers**	2 lab technicians
	4 midwives
	6 auxiliary midwives
**Community health workers**	4 community care workers
	7 drug shop managers
**NGO and private**	2 NGO managers
	1 midwife
	1 lab technician
	1 pharmacist
**Facility manager**	7 (Primary health care center)
**Community**	35 pregnant women
	4 traditional healers

The healthcare providers included doctors, midwives, nurses, laboratory personnel, and pharmacists. Participants were purposively selected to capture a range of perspectives across different actors with varying roles and responsibilities. In each health facility, five pregnant women were approached as they queued to receive antenatal services and informed about the study. For those who consented, the interview was held at the end of their visit. Although we initially planned to have focus group discussions (FGDs) with pregnant women, the number of women available for the FGDs was too small because data collection occurred during the rainy season.

### Data collection

Data were collected using interview guides that were adapted for each profile of respondent. The interview with pregnant women explored experiences of ANC, satisfaction with ANC, knowledge and perceptions of sexually transmitted infections (STIs) including syphilis, perceptions of existing point-of-care tests, opinion on the introduction of additional test. We sought to find out key informants’ perception of ANC, management of STI during pregnancy, knowledge and perceptions of syphilis, barriers and constraints which affect the effective delivery of maternal syphilis screening, organizational and managerial issues, experience with point-of-care tests, and introduction of a rapid diagnostic test (RDT) for syphilis screening.

Data tools were pre-tested and appropriate modifications made before the final data collection. Data collection was conducted by the first author and two research assistants who are familiar with qualitative studies and have a social sciences background. Research assistants were trained on the study objectives, data collection tools, and processes before embarking on field data collection. Interviews with health providers, district managers, facility managers, and NGO managers were conducted in French while those with pregnant women, traditional healers and community workers were conducted in Mooré the local language. Appointments were made with community health workers, traditional healers, health providers, district managers, facility managers and NGO managers. All interviews were recorded using a digital recorder and files downloaded to a laptop the same day. Transcription was done by two transcribers. Interviews in Mooré were translated into French and transcribed.

### Data analysis

Interviews were transcribed into a text program and then uploaded on Nvivo software. An analytical grid of key themes was developed based on the list of possible barriers in our conceptual framework, the objectives of the research and familiarization with the first few transcripts. Additional themes that emerged during the process of re-reading of transcripts were coded. Thematic content analysis was employed to systematically analyze the content of each theme.

### Ethical issues

Ethical clearance was obtained from the University of Western Cape, the National Ethics Committee for Health in Burkina Faso and the Ethics Committee Review of the WHO. In addition, the study team obtained permission to conduct the research from the District authorities. Written informed consent was obtained from all participants.

## Results

Although the guidelines on the management of STIs recommend syphilis screening for all pregnant women, we found no information on the proportion of pregnant women routinely tested for syphilis at district, regional and national level. Our study findings highlight considerable weaknesses within operational systems for syphilis screening. In tracking a woman's journey from antenatal care (ANC) through to laboratory, the study documented several barriers at health provider and community levels.

### Barriers at health provider level

The first barrier to routine syphilis screening among pregnant women was related to providers’ perception that syphilis in pregnancy was not an important issue relative to other diseases. In addition, health workers also felt that syphilis prevalence was low because most women who undertook the test were seronegative. As one auxiliary midwife who had worked in an urban facility since 2009 stated, “I have never found a positive test, all were negative.” Related to this, some health workers felt that syphilis was more prevalent in urban areas and thus, screening was more systematic in urban-based facilities. One district manager noted “For syphilis screening it is not really systematic and I know that in urban facilities health workers prescribe it to all women but at rural facilities it is not systematic.” Overall, we noted an absence of interventions and information on maternal syphilis in the district.

The second barrier to routine syphilis screening among pregnant women was related to the availability of screening equipment, which was particularly a challenge for rural facilities. One facility manager in a rural-based facility noted that, “I do not systematically prescribe syphilis test because we have no laboratory here.” A mapping of thefacilities in the district indicated that three facilities (one public and two private) offered the syphilis test. All three facilities were urban-based. The public facility hosts the laboratory of the regional hospital and performs rapid plasma reagin (RPR) and Treponema pallidum particle agglutination assay (TPHA) tests. Between August 2011 and August 2012, this facility had performed 279 RPR tests and 260 TPHA tests. Among the two private that offered screening, one was a pharmacy offering a point of care test, while the second was a faith based facility offering the RPR and TPHA test. At the private pharmacy, only four clients had requested a syphilis test between June 2011 and June 2012. At the faith based facility, 50 RPR tests and 10 TPHA tests were performed between August 2011 and August 2012. The third barrier was related to health workers’ inability to communicate the need for syphilis screening to pregnant women. Health workers noted that it was difficult to convince women about the importance of screening for syphilis. This challenge was partly related to the need to collect multiple blood samples from women for an HIV test, as part of the PMTCT program, as well as for the syphilis test. According to one health worker, women did not understand the need for multiple blood tests: “when we do an HIV test, you get a blood sample. We said that they need to go to the laboratory and have another blood test to know if they have other diseases, they said no, it is the same blood you got here and tested it is not necessary to have another sample.” Health workers acknowledged that they did not explain the importance of some of the examinations. For example, one manager in an urban-based facility noted that “Most of the time it is a lack of communication at our level, we do not tell to the women the importance of some exams, why this prescription…” The narratives from pregnant women corroborated this observation. One pregnant woman in a rural area stated “One day the health worker took blood from my left finger but I did not know if it is HIV test or not…) I don't know because until now they have said nothing.” The poor communication between health workers and pregnant woman may be related to the lack of routine training as the health workers stated that they had no specific training, except for a course on syphilis management during their professional training.

A fourth barrier was the fragmentation of services in a setting where geographic distance was already a barrier. Often women have to be referred to an external laboratory for the syphilis screening. According to health workers, many women live in rural areas and have to travel long distances to health facilities that offer screening services. One health worker commented, “we observe that most women are from villages around Kaya, they walk from their house to our facility and we ask them to do the test the day after. The distance from their house to the laboratory is same to our facility. Thus the majority do not go.” We observed that traveling from the nearest urban primary health facilities to the public laboratory would take about 1 hour by foot or 20 minutes by bicycle. Due to this situation, one urban health center had a lab technician who came to the facility to collect blood samples, but few women did the test.

### Barriers at community level

The cost of the syphilis test was reported to be a barrier for many women. At the public laboratory, RPR and TPHA cost the equivalent of USD 2-3. The point of care test cost more in the pharmacy (USD 3) compared with the faith based facility (USD 2). Although the cost of syphilis tests in the public sector is subsidized by the government, many women are not screened because of the cost of the test. One auxiliary nurse stated “There are women who keep the exam prescription until delivery because they said that they have no money for the exams.”

Our findings also indicate that a pregnant woman's husband or partner plays a key role in the decision to be screened for syphilis. Due to exemption of fees, women do not carry a lot of money when they go for their ANC visit. When they receive a prescription for additional medical examinations, such as the syphilis test, they have to go back home and get money from their husband. Sometimes, women need approval from their husbands as illustrated by this quote from a midwife in an urban facility, “sometimes, until the delivery they (women) kept the prescription in their health card, when you ask them why, they explain that they gave it to the head of the family but he did nothing.”

Findings also show that poor knowledge about syphilis was also a potential barrier to testing. For example, although many women could describe the symptoms of sexually transmitted infections (STIs) (itches, pimples, and vaginal discharge), many were unaware about syphilis or the consequences of untreated syphilis for the mother and child.

Finally, perceptions about syphilis also affected screening rates. In particular, the stigma surrounding sexually transmitted diseases was noted as a barrier to screening particularly in certain settings like pharmacies. One facility manager noted “the pharmacy advertised and gave the prices but you know women, it is difficult for them to go to the pharmacy and do an exam related to sex. They prefer to go to the laboratory of the hospital if they have money because it is a public service.”

## Discussion

Syphilis screening is recommended for premarital tests and during pregnancy [14]. Although a policy that promotes syphilis screening in pregnant women exists in Burkina Faso, screening is very limited. Our findings identified several barriers to the uptake of syphilis screening among pregnant women in Burkina Faso.

Syphilis testing is largely dependent on the availability of adequate laboratory facilities [19]. However, our results suggest that the fragmentation of services is key barrier to the uptake of syphilis screening. Health workers often have to refer women to external laboratories and many women, particularly those living in rural areas, have to travel long distances to access these laboratories. Other studies have also reported that long distances to screening facilities are associated with delay or failure to screen [20, 21]. Our findings suggest the need to introduce a “one-stop” service point that including ANC, PMTCT and syphilis testing.

As highlighted in previous studies [22, 23], we found that low motivation of healthcare workers to prescribe syphilis screening also contributes to low screening. Although the need for continued antenatal screening for syphilis may be questionable in areas with low prevalence [24], health workers in the current study were not aware about the relatively high prevalence of syphilis in their district. Consequently, some health workers failed to prescribe the test. Trepka et al [25] also found that a lack of provider awareness of the prevalence of syphilis was associated with inadequate provision of screening test in the United States. The absence of interventions to increase syphilis screening and the lack of information on maternal syphilis in the district shows also the low prioritization of the problem. Efforts to increase awareness about syphilis are therefore warranted in order to enhance syphilis screening levels.

The relatively high cost of screening, despite government subsidies, also prevents pregnant women from being screened for syphilis. The cost for testing was observed to range between $2 and $3 USD, a prohibitive cost in a country where 73% of population lives on less than $2 a day [26]. The cost of screening is, therefore, a significant deterrent for many women particularly those who are financially dependent on their husband or partner. Women's financial dependency means that pregnant women's husbands or partners play a key role in the decision to be screened. Similar findings have been highlighted in previous studies [27, 28] and underscore the need for male involvement in efforts to increase the uptake of syphilis screening among pregnant women.

Lack of knowledge about syphilis in the community was identified as a reason for not being screened. Most respondents at community level do not know the symptoms of syphilis nor its serious consequences for the unborn and born child. This misperception may be due to the lack of differentiation between STIs [29]. Most of STIs are recognized through symptoms and respondents do not realize that a STI could be asymptomatic. Community may also not perceive syphilis to be a problem because of its lack of visibility [30]. Low knowledge about syphilis might therefore pose a barrier to screening since pregnant women do not perceive the benefit of testing particularly for asymptomatic infections. As reported in a recent meta synthesis, many pregnant women did not feel the need to seek professional care when there is nothing wrong with their pregnancy [31]. Efforts to enhance awareness of syphilis and other STIs are therefore recommended.

Our study findings should be interpreted in light of several limitations. First, because of the exploratory nature of the study, we relied on qualitative methods and therefore our findings cannot be generalized to the larger population. Second, community's perceptions reflected mostly health services users. However, study findings highlight potential barriers to the uptake of syphilis screening. Further research using a more representative sample is warranted.

## Conclusion

Our study suggests that barriers such as distance to health facilities, cost of testing, and knowledge about syphilis among health workers and communities may limit screening levels and hinder the implementation of syphilis screening during pregnancy as recommended in national guidelines. Pregnant women often weigh the benefits of syphilis screening against the high direct and opportunity costs. Our results have several implications for efforts to improve screening levels. First, communication between health workers and clients needs to be improved in order to facilitate the acceptability of the test. Second, the introduction of point of care testing for syphilis during ANC may improve coverage of antenatal syphilis screening.
